# Microbial Diversity and Infection Burden in Central Nervous System (CNS) Shunt and Drain Devices: A Prospective Observational Study

**DOI:** 10.7759/cureus.95294

**Published:** 2025-10-24

**Authors:** Sheetal Agarwal, Prashant Gupta, Vimala Venkatesh, Chhitij Srivastava

**Affiliations:** 1 Department of Microbiology, Hind Institute of Medical Sciences, Barabanki, IND; 2 Department of Microbiology, King George's Medical University, Lucknow, IND; 3 Department of Neurosurgery, King George's Medical University, Lucknow, IND

**Keywords:** central nervous system (cns), device-associated infection (dai), external ventricular drain (evd), ommaya reservoir, ventriculoperitoneal (vps)

## Abstract

Introduction: Device-associated central nervous system infections are a subgroup of healthcare-associated infections that occur when either a temporary or permanent surgically implanted device becomes a source of infection. These devices are used to treat raised intracranial pressure due to hydrocephalus. These surgically implanted devices may become a source of infection due to colonization of microbes or contamination from the hospital environment. To determine the diversity of organisms involved in infection using MALDI-TOF-based identification, to assess the antimicrobial profiles of isolated organisms, and to calculate the rate of infection of these devices in our institution, we conducted this study.

Methods: This prospective observational study was conducted over one year at the Departments of Microbiology and Neurosurgery in a tertiary care teaching institute in North India. All patients with cerebrospinal fluid (CSF) drainage devices, such as external ventricular drains (EVDs), ventriculoperitoneal shunts (VPS), Ommaya reservoirs, and lumbar drains, with clinical suspicion of infection in the form of meningitis, meningoencephalitis, or ventriculitis, were included.

CSF samples were cultured on 5% sheep blood agar, chocolate agar, and MacConkey agar. Organisms from positive cultures were identified using matrix-assisted laser desorption/ionization time of flight (MALDI-TOF). Antimicrobial susceptibility was determined by the Kirby-Bauer disk diffusion method on Mueller-Hinton agar plates after preparing a 0.5 McFarland inoculum. The results were interpreted as per the Clinical and Laboratory Standards Institute guidelines, M100. Device-associated infection (DAI) rates were calculated monthly.

Results: A total of 107 patients were included. The majority of enrolled patients were men (male-to-female ratio: 1.97:1). Adults comprised 64.5% followed by infants (22.43%) and children (13.08%).

Most patients presented with vomiting (41.12%), headache (40.19%), and altered sensorium. VPS was the most commonly used device (65.4%), followed by EVD (30.14%). Among the 231 CSF samples received, 31 from 17 patients showed microbial growth. *Klebsiella pneumoniae *was the most frequently isolated organism (51.6%), followed by *Pseudomonas aeruginosa* (19.35%) and *Acinetobacter baumannii* (9.68%). Device-wise distribution showed that *K. pneumoniae *was the predominant organism isolated from EVD (42.8%), VPS (55.55%), and Ommaya reservoirs (83.33%). Infections were more common during hot and humid months, with the highest DAI rate in September (14.28%). Of the 107 patients, 83 were discharged, 15 died, and nine were lost to follow-up. Among the 17 patients, whose CSF cultures had shown growth of micro-organisms, 14 were discharged, two died, and one left against medical advice.

Conclusion: Our study highlights that DAIs are more prevalent with EVDs and are predominantly caused by Gram-negative organisms, with *K. pneumoniae *being the most frequently isolated. The susceptibility patterns underscore the need for regular antimicrobial surveillance and judicious antibiotic use to prevent the development of drug resistance. The seasonal variation in infection rates highlights the importance of stricter infection control measures during the high-risk months in hot and humid environments. Thus, early diagnosis, rational antibiotic use, and stringent infection control practices are essential for improving patient outcomes in neurosurgical settings.

## Introduction

Device-associated central nervous system (CNS) infections are a major subgroup of healthcare-associated CNS infections that occur when a surgically implanted device, whether temporary or permanent, becomes a source of infection. These devices are used to treat raised intracranial pressure (ICP) due to hydrocephalus.

An external ventricular drain (EVD) is the most commonly used temporary device. It is also known as a ventriculostomy catheter. It is a silicone catheter used to treat hydrocephalus, monitor ICP, and drain intraventricular hemorrhage. Although sterile, EVD can become contaminated during placement, handling, or maintenance [1].

A lumbar drain is also a temporary device for cerebrospinal fluid (CSF) drainage. It is used to control refractory intracranial hypertension, preoperative evaluation of normal pressure hydrocephalus, drainage of infectious or bloody CSF, intraoperative cerebral relaxation, and prevention of complications secondary to aneurysmal subarachnoid hemorrhage [2].

Ommaya reservoir is a small dome-shaped reservoir connected to an intraventricular catheter. It is placed subcutaneously in the scalp. It is used to deliver regular intraventricular therapy and also collect a sample of CSF without the need for serial lumbar punctures [3].

Ventriculoperitoneal shunts (VPS) are the most commonly used permanent devices. They divert CSF from the CNS ventricular system to the peritoneal cavity. They are used to treat chronic hydrocephalus [4].

These surgically implanted devices may become a source of infection due to colonization of microbes or contamination from the hospital environment. Few studies from the US and Europe suggest the rate of infection of these devices to be 2%-22% [1]. There is a wide variation in the observed rate of infection, as it depends on device utilization, handling, and the need for its manipulation at various institutions. This study was conducted to determine the microbial profile and antibiotic susceptibility patterns, as well as to calculate the rate of CNS device-associated infections (DAIs) in our institution.

## Materials and methods

This prospective observational study was conducted over a period of one year at the Departments of Microbiology and Neurosurgery in a tertiary care teaching institute in North India. All patients with CSF drainage devices, such as EVD, VPS, Ommaya reservoir, or lumbar drain, who had clinical suspicion of infection in the form of meningitis, meningoencephalitis, or ventriculitis, were included in the study. Both pediatric and adult age groups of patients admitted to Neurosurgery with CSF drainage devices were included.

Case definition

The device-related infection rate is defined as follows: 1) positive CSF culture result plus clinical symptoms or CNS pleocytosis/cell count increase, or 2) in the case of negative CSF culture, clinical symptoms and CNS pleocytosis/cell count increase.

Ethical approval

The Institutional Ethics Committee approval was obtained (Ref. code: 97th ECMI II B-Thesis/P74).

Specimen collection and transport

CSF sample of amount 1-5 mL was collected from the device in a sterile screw capped tube at the Neurosurgery Department and was immediately transported to the Microbiology Laboratory (within one hour) and processed as per standard protocols.

Processing of the specimen

The gross appearance of CSF-like volume was noted. The sample was cultured on 5% sheep blood agar (SBA), chocolate agar (CA), and MacConkey agar (MA). SBA and CA were incubated in a candle jar, and MA was incubated aerobically at 37℃ for 48-72 hours. Cell count was done using a Neubauer hemocytometer (Shanghai Qiujing Biochemical Reagent Instrument Co., Ltd., Shanghai, China). It has a 3 mm counting grid. Each grid has nine square subdivisions of width 1 mm. Cell count was done manually on an undiluted sample. If CSF appeared turbid or cloudy, a 1:20 dilution was made using 0.05 mL of CSF and 0.95 mL of Turk solution. The cells were counted in four corner squares. The specimen was centrifuged at 3,500 rpm for 15-20 minutes, followed by direct Gram staining and wet mount examination to look for pus cells, RBCs, and microorganisms. Biochemical analysis was also performed.

Identification and antimicrobial susceptibility testing

Organisms from positive cultures were identified using matrix-assisted laser desorption/ionization time of flight. It is an analytical technique in which samples are ionized into charged molecules, and the ratio of the mass to charge (m/z) is measured by determining the time the ions take to travel to a detector at the end of a time-of-flight tube. The resulting spectra are compared to a database of spectra from known organisms.

Antimicrobial susceptibility pattern of the isolates was determined by the Kirby-Bauer disk diffusion method on Mueller-Hinton agar plates after preparing a 0.5 McFarland inoculum. Susceptibility test discs of HiMedia Laboratories Pvt Ltd, Mumbai, India, were used. A maximum of six discs was applied on a 100 mm diameter plate. The results were interpreted as per the Clinical and Laboratory Standards Institute guidelines, M100 [5].

The antibiotics used for antibiotic susceptibility of Gram Negative organisms were amikacin (30 mcg), gentamicin (10 mcg), trimethoprim-sulfamethoxazole (25 mcg), piperacillin-tazobactam (100 mcg), aztreonam (50 mcg), meropenem (10 mcg), imipenem (10 mcg), tobramycin (10 mcg), amoxyclav (30 mcg), ampicillin (10 mcg), ceftriaxone (30 mcg), cefepime (30 mcg), and ceftazidime (30 mcg). The antibiotics used for antibiotic susceptibility of Gram-positive organisms were penicillin (10 units), amikacin (30 mcg), trimethoprim-sulfamethoxazole (25 mcg), vancomycin (30 mcg), optochin (5 mcg), chloramphenicol (30 mcg), bacitracin (0.04 U), gentamicin (10 mcg), and high-level gentamicin (120 mcg).

DAI rate was calculated on a monthly basis using the following formula:

$\text{DAI rate} = \frac{\text{Number of device-associated infections at a specific site} \times 1,000} {\text{Total number of device days}}$.

Here, device days are the total number of days of exposure to the device (EVD, Shunt, Ommaya, Lumbar drain) by all patients in the selected population during the selected time period.

Statistical analysis

The data were entered on the Excel sheet (Microsoft Corporation, Redmond, WA), and analysis was done using Statistical Package for the Social Sciences (SPSS) version 22.0 (IBM Corp., Armonk, NY). A significance level of p < 0.05 was considered statistically significant. Please note that only the SPSS software was used. No other tools were used in this study.

## Results

A total of 107 patients (n) were studied, while the total number of device insertions (N) is higher, as some patients required more than one device placement. The majority of patients enrolled were men (male-to-female ratio is 1.97). Adults comprised 64.5% followed by infants (22.43%) and children (13.08%). Most of the patients presented with vomiting (41.12%), headache (40.19%), and altered sensorium (35.51%) (Table 1).

**Table 1:** Signs and symptoms of patients at the time of admission ^*^p value of <0.05 was considered statistically significant

Signs and symptoms	n (%)	p value
Vomiting	44 (41.12%)	X = 55.98, p < 0.0001^*^
Headache	43 (40.19%)	
Altered sensorium	38 (35.51%)	
Blurring of vision	10 (9.35%)	
Loss of consciousness	5 (4.67%)	
Seizures	19 (17.76%)	
Neurological deficit	19 (17.76%)	
Increase in head size	22 (20.56%)	
Loss of developmental milestones	5 (4.67%)	
History of road traffic accidents	4 (3.74%)	

The most commonly used device was VPS (65.4%), followed by EVD (30.14%), as shown in Table 2.

**Table 2:** Distribution of patients according to device used (n = number of patients with device) ^*^p value of <0.05 was considered statistically significant

Device used	n (%)	p value
External ventricular drain	41 (30.14%)	X = 110.3, p < 0.0001^*^
Ventriculoperitoneal shunt	89 (65.4%)	
Ommaya placement	4 (2.9%)	
Lumbar drain	2 (1.4%)	
Total	136 (100.0%)	

CSF samples at both the device insertion and the onset of the first symptom could be collected in only 76 cases. Mean ± SD of protein, glucose, total leukocyte count, neutrophils, and lymphocytes were compared at both time intervals. On applying the unpaired t-test, the p value was not found to be significant (>0.05).

Among the 231 CSF samples received for culture, 200 (86.6%) were sterile, while 31 (13.4%) from 17 patients yielded microbial growth. The Gram-negative isolates included *Klebsiella pneumoniae *(16), *Pseudomonas aeruginosa* (six),* Acinetobacter baumannii *(three), and *Escherichia coli *(one). The Gram-positive isolates were *Staphylococcus aureus*, *Staphylococcus hominis, Staphylococcus sciuri, Streptococcus infantarius,* and* Enterococcus faecium *(one each). *K. pneumoniae* was the most commonly isolated pathogen (51.6%), followed by *P. aeruginosa *(19.35%) and *A. baumannii* (9.68%). Device-wise distribution showed that *K. pneumoniae* was the predominant organism isolated from EVD (42.8%), VPS (55.55%), and Ommaya reservoirs (83.33%) (Table 3).

**Table 3:** Distribution of organisms according to the device used (n = 31)

Organism	Device used				Total
	EVD	VPS	Ommaya reservoir	Lumbar drain	
Klebsiella pneumoniae	6	5	5	0	16
Pseudomonas aeruginosa	3	1	1	1	6
Acinetobacter baumannii	2	1	0	0	3
Escherichia coli	1	0	0	0	1
Staphylococcus aureus	1	0	0	0	1
Staphylococcus hominis	1	0	0	0	1
Staphylococcus sciuri	0	1	0	0	1
Enterococcus faecium	0	0	0	1	1
Streptococcus infantarius	0	1	0	0	1
Total	14	9	6	2	31
Percentage	45.16%	29%	19.35%	6.45.0%	100.0%

The susceptibility pattern of Enterobacterales (n = 17) is shown in Table 4.

**Table 4:** Sensitivity pattern of drugs among Enterobacterales ^*^p value of <0.05 was considered statistically significant

S. no.	Antibiotic	Total specimens	Sensitive, n (%)	Resistant, n (%)	p value
1	Amikacin	17	8 (47.05%)	9 (52.9%)	X = 0.1176, p = 0.7316
2	Gentamicin	17	3 (17.64%)	13 (82.35%)	X = 11.81, p = 0.0006^*^
3	Trimethoprim-sulfamethoxazole	17	13 (76.47%)	4 (23.52%)	X = 9.529, p = 0.0020^*^
4	Piperacillin-tazobactam	17	4 (23.52%)	13 (76.47%)	X = 9.529, p = 0.0020^*^
5	Cefepime	17	1 (5.88%)	16 (94.11%)	X = 26.47, p < 0.0001^*^
6	Aztreonam	17	5 (29.4%)	12 (70.58%)	X = 5.765, p = 0.0164^*^
7	Meropenem	17	10 (58.827%)	7 (41.17%)	X = 1.059, p = 0.3035
8	Imipenem	17	9 (52.64%)	8 (47.05%)	X = 0.1176, p = 0.7316
9	Tobramycin	17	6 (35.29%)	11 (64.70%)	X = 2.941, p = 0.0863
10	Amoxyclav	17	4 (23.52%)	13 (76.27%)	X = 8.529, p = 0.0020^*^
11	Ampicillin	1	1 (100%)	0 (0.00%)	X = 2.000, p = 0.1573
12	Ceftriaxone	17	10 (58.82%)	7 (41.17%)	X = 1.059, p = 0.3035

**Figure 1:**
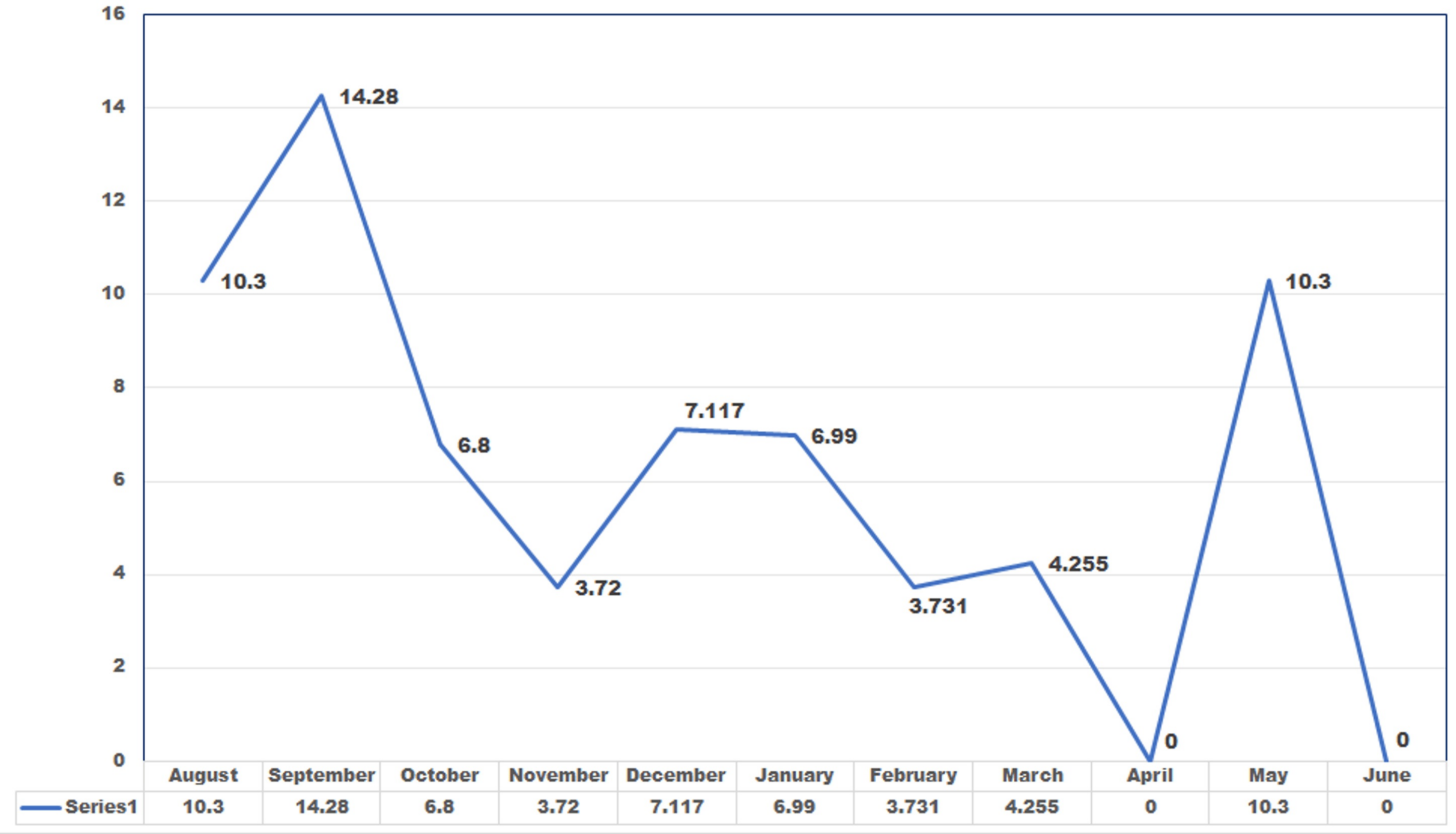
Month-wise device infection rate of CSF drainage devices during the study period CSF: cerebrospinal fluid

Since ampicillin is intrinsically resistant in *K. pneumoniae*, it was reported only in a single *E. coli *isolate that was sensitive. DAI rate was maximum during September (14.28%), followed by August and May (10.3% each), as shown in Figure 1.

Of the total 107 patients, 83 (77.57%) were discharged, 15 (14.02 %) died, and nine (8.41 %) were lost to follow-up as they left against medical advice (LAMA). Among the 17 patients, whose CSF cultures had shown growth of micro-organisms, 14 were discharged, two died, and one went LAMA.

## Discussion

In our study, men were affected nearly twice compared to women (1.97:1). A study by Deshmukh and Yadav [6] in Government Medical College, Maharashtra, also found the male-to-female ratio in patients with hydrocephalus to be 1.7:1. In our study, 61.6 % patients were adults, which is contradictory to other studies done worldwide which says hydrocephalus is more common in pediatric age group, which is mainly due to birth defects. A review done by Isaacs et al. says that 77% of hydrocephalus patients are pediatric, 13% elderly, and only 10% are the adult population [7]. This discrepancy in our study is because our hospital has a trauma emergency unit where we frequently receive trauma head injury cases. And since we enrolled the patients mainly from neurosurgery, we had a larger number of adult patients with hydrocephalus. Neurosurgery for pediatric patients is performed, but they are soon transferred to pediatric ICUs or wards, which is why they cannot be enrolled.

Most of the patients complained of vomiting, headache, altered sensorium, and blurring of vision at the time of admission to the hospital, as the p value is statistically significant <0.0001, as shown in Table 1. These complaints are mainly seen in patients with CNS infections in our study.

A study done by Polis et al. states that the most common surgical treatment for hydrocephalus is the implantation of VPS [8]. It is similar to our study, as in most patients, shunts are used to treat hydrocephalus. The distribution of device utilization was statistically significant (p < 0.0001), as seen in Table 2, highlighting a consistent preference for VPS in clinical scenarios in our study.

Although VPS was used in 89 patients in our study, six patients had both right and left shunts used. Therefore, there were a total of 95 episodes of VPS. Among these 95, CSF culture was positive in eight (8.42%) episodes. Among these eight, two patients had previously undergone Ommaya reservoir placement, which became infected. After the removal of the Ommaya, a shunt was placed, but it subsequently became infected.

Another patient initially had an infected EVD, which was replaced with VPS, but it also got infected and was replaced by an Ommaya reservoir. The reason for these recurrent infections of new devices could be biofilm formation in the ventricles. Bacteria form biofilms and attach to the surface of implanted devices to cause infections. Biofilms are able to evade the host immune defense and are resistant to antimicrobial treatment [9].

CSF cultures from 87 shunts (91.58 %) were sterile. In these patients, there was no infection, or the organisms were noncultivable. There could be a possibility of anaerobic infection, as anaerobic cultures were not performed in our study. Moreover, the patient was on prophylactic antibiotic therapy, which could be a probable reason for the CSF cultures being negative.

In our study, Gram-negative organisms such as *K. pneumonia, P. aeruginosa*,* *and *A. baumannii *were more commonly isolated from VPS. Among the Gram-positive bacteria, one *Streptococcus infantarius *and one *Staphylococcus hominis* were isolated, as shown in Table 3. In a study from North India, *A. baumannii* was the most common causative organism isolated from shunt infections, followed by *K. pneumoniae *[10]. However, a study by Gutierrez-Murgas and Snowden in the USA found that shunt infections were more commonly caused by normal skin flora, such as coagulase-negative Staphylococci*, S. aureus, *and* Propionibacterium acnes*, which may be introduced at the time of surgery. However, they also reported a few Gram-negative organisms [11]. It is contrary to our study, which found that shunt infections were caused more by Gram-negative bacteria.

Trauma cases need daily hygiene maintenance in the form of body cleaning, device insertion site cleaning, and disinfection. Due to excessive patient load in a hospital, daily cleaning and disinfection of patient body surfaces may not be proper, which can result in increased infection by Gram-negative organisms. Other means of infection could be hematogenous spread from a distant focus or contiguous infection from wounds or perforated gut.

EVD was used in 41 patients. In one patient, two EVDs were used (both in the left and right ventricles). Therefore, there were a total of 42 episodes of EVD. Thirty-one percent of EVDs grew organisms on culture, with Gram-negative pathogens again outnumbering Gram-positive organisms, as depicted in Table 3. Our finding is similar to a study done in South India, where *K. pneumoniae* was the most common causative agent, followed by* A. baumannii* and *P. aeruginosa *[12].

Among all the devices used in our study, EVDs were found to be most commonly infected, as shown in Table 3. This generates the need for infection prevention care bundles for EVD and other CSF drainage devices. A 2025 prospective study demonstrated that implementation of standardized insertion and maintenance bundles significantly reduced infection rates in EVDs, reinforcing the importance of evidence-based preventive strategies [13]. We suggest inspecting the EVD insertion site for signs of CSF leak or local skin infection, and disinfecting daily with povidone-iodine solution. We also suggest changing the external collecting bag of the drainage system daily using sterile gloves and keeping the level of the collection bag below the bed level to prevent any backflow of CSF.

The Ommaya reservoir in our study was used in only four episodes. A seven-year-old patient had an Ommaya replaced thrice as they all got infected with multidrug-resistant (MDR) *K. pneumonia*. Ultimately, intrathecal colistin was administered, but the patient succumbed to the infection. *K. pneumoniae* is a known MDR pathogen and also has the capability to form biofilm.

In our study, lumbar drains were used in only one patient, where two lumbar drains were used as the first one got infected. First, a VPS was inserted, which became infected by *P. aeruginosa.* It was replaced by a lumbar drain, which also got infected by the same organism. It was again replaced by another lumbar drain. The CSF culture from the second lumbar drain showed growth of *Enterococcus faecium*. It was again replaced by the Ommaya reservoir, which showed growth of *P. aeruginosa.* The patient could not be saved and succumbed. A study by Liang et al. [14] from China showed that 84.6% infections in lumbar drains are caused by Gram-positive bacteria and 15.38% by Gram-negative bacteria.

For Enterobacterales(n = 17), the most sensitive drug was trimethoprim-sulfamethoxazole with a p value of 0.0020, while the most resistant drugs were cefepime (p < 0.0001), gentamicin (p = 0.0006), piperacillin-tazobactam, and amoxyclav (p = 0.0020), followed by aztreonam (p = 0.0164), as shown in Table 4. For *P. aeruginosa* (n = 6), the most sensitive drugs were amikacin, piperacillin-tazobactam, imipenem, and ceftazidime (p = 0.0005), followed by meropenem and gentamicin (p = 0.0209). For Staphylococcal species (n = 3), sensitive drugs were amikacin, gentamicin, and cefoxitin (p = 0.0143).

As statistical significance is observed in all these cases, our study suggests that identifying causative organisms and understanding their sensitivity patterns through antibiograms are necessary for choosing empiric therapy and combating emerging antibiotic resistance.

For *Streptococcus infantarius* (n = 1), sensitive drugs were chloramphenicol, trimethoprim-sulfamethoxazole, vancomycin, and optochin, whereas resistant drugs were penicillin, piperacillin-tazobactam, and bacitracin. For *Enterococcus faecium* (n = 1), the sensitive drug was vancomycin, while the resistant drugs were ampicillin and high-level gentamicin. As we found only a single isolate of both *S. infantarius *and *E. faecium,* we could not comment on the susceptibility pattern of these two species.

In a study of VPS infections [15], the most susceptible drugs for Enterobacteriaceae and *P. aeruginosa* were piperacillin-tazobactam, amikacin, and meropenem; and piperacillin-tazobactam and meropenem for *A. baumannii*. For Staphylococcal species, linezolid and vancomycin were the susceptible drugs.

The device-related infection rate in our study was highest in September (14.28%), followed by 10.3% in both August and May, as shown in the line diagram (Figure 1). North India has a hot and humid climate from July to September, and this climate is favorable for the growth of micro-organisms. This could be the reason for the increased infection rate in these months. A three-month prospective study from South India reported the infection rate of EVD to be 19.4 per 1000 EVD days in their institute [16]. Similarly, a 2025 European surveillance report confirmed seasonal variation in device-related infections, correlating higher infection rates with warm and humid climates, which parallels our findings [17].

The study has a few limitations, such as the inability to perform anaerobic culture. Moreover, very few cases with lumbar drain and Ommaya reservoir could be enrolled; therefore, the infection rate of individual devices could not be determined.

## Conclusions

Infections associated with indwelling CNS devices are a cause of significant challenge in neurosurgery. Our study highlights that DAIs are more prevalent with EVDs and are predominantly caused by Gram-negative organisms, with K. pneumoniae being the most frequently isolated. Infection control can be strengthened through proper aseptic insertion and adherence to standardized care bundles during device maintenance. Regular staff training will significantly reduce infection rates. Moreover, microbiological surveillance to guide empirical therapy is necessary to avoid the emergence of resistance. The seasonal variation in infection rates highlights the importance of stricter infection control measures during high-risk months in hot and humid environments. Thus, early diagnosis, rational use of antibiotics and stringent infection control practices are essential in improving patient outcomes in neurosurgical settings.
